# The influence of iconicity and autistic traits on novel word learning: a cross-cultural investigation

**DOI:** 10.1098/rsos.242161

**Published:** 2025-03-26

**Authors:** Vijayachandra Ramachandra, Kairi Sugimoto, Kelly Ziskind, Ark Verma, Irfan Ahmad, Mahayana Godoy, Katsumi Watanabe

**Affiliations:** ^1^ Department of Communication Sciences and Disorders, Marywood University, Scranton, PA, USA; ^2^ Faculty of Science and Engineering, Waseda University, Shinjuku-ku, Tokyo, Japan; ^3^ Clinical Fellow in Speech-Language Pathology, Theracare, Hackensack, NJ, USA; ^4^ Department of Cognitive Science, Indian Institute of Technology Kanpur, Kanpur, Uttar Pradesh, India; ^5^ Center for the Humanities, Languages and Arts, Federal University of Rio Grande do Norte, Natal, Brazil

**Keywords:** iconicity, autistic traits, sound symbolism, novel word learning, autism spectrum quotient

## Abstract

The effects of iconicity and autistic traits on novel word learning were investigated through an online experiment involving 1481 healthy adult participants aged between 18 and 40 years from four countries: Brazil (*N* = 261), India (*N* = 416), Japan (*N* = 493) and the USA (*N* = 311). Participants completed a bouba–kiki-based word learning task, viewing novel images paired with either iconic names (congruent condition) or arbitrary names (incongruent condition). Word recognition was assessed using a three-alternative forced-choice procedure, and autistic traits were measured with the autism spectrum quotient (AQ). Results showed a significant benefit of iconicity across all countries, with better performance in the congruent condition. While a linear mixed model revealed no significant effect of AQ on bouba–kiki scores overall, a country-specific analysis found a weak but significant positive correlation between AQ scores and bouba–kiki performance in Japanese participants. This country-specific finding should be interpreted cautiously and warrants further exploration. Overall, the findings demonstrate the robustness and universality of the bouba–kiki effect on word learning across both Western and Eastern cultures. However, the relationship between autistic traits and iconicity was not consistent across all countries and may depend on cultural factors. Further research is needed to explore this in more detail.

## 1. Introduction

Although the majority of words in a language are generally ‘arbitrary’—meaning there is no connection between a word and the object it refers to [1]—many words in languages such as sub-Saharan African, Australian Aboriginal and many south Asian languages, display sound symbolism or iconicity, where there is a resemblance between features of a word and its meaning [2,3]. Some words in English such as ‘meow’, ‘splash’, ‘trill’, ‘moo’, etc., which resemble some aspects of the referent are also iconic. Another example of iconicity is *size sound symbolism*, where a high front vowel like /i/which is produced with a small mouth opening refers to something small (e.g. teeny) and the low back vowel like /a/ which is made with a large mouth opening refers to something large (e.g. the word large) [4]. English words such as gleam, glitter and glow (all with a prefix ‘gl’), which all refer to ‘light’, reflect another form of iconicity known as systematicity. Systematicity refers to the phenomenon where certain sounds or groups of sounds systematically relate to specific meanings [2–4].

Several studies indicate that children are sensitive to the iconic properties of their languages [5–7], and the initial words spoken by young children tend to be more iconic than those acquired later in life [8,9]. These findings support Imai & Kita’s [10] bootstrapping theory, which posits that iconicity can provide learners with a foundation for not only learning iconic words but also other aspects of language. The early vocabulary of young children in several languages is relatively more iconic than the vocabulary learned later in lexical development [8,11,12]. Finally, adults use them more often while conversing with young children than adults [9]. Neuroimaging studies have shown that iconic words that are effective, where there is a direct link between the sounds embedded within words and their emotional meanings, may be advantageous because they recruit additional neural structures subserving emotions like the left amygdala [13]. A study conducted by Meteyard *et al*. [14] showed the benefits of iconicity in facilitating language in adults with aphasia, a language disorder caused generally by strokes.

### 1.1. The bouba–kiki task

**Figure 1.**
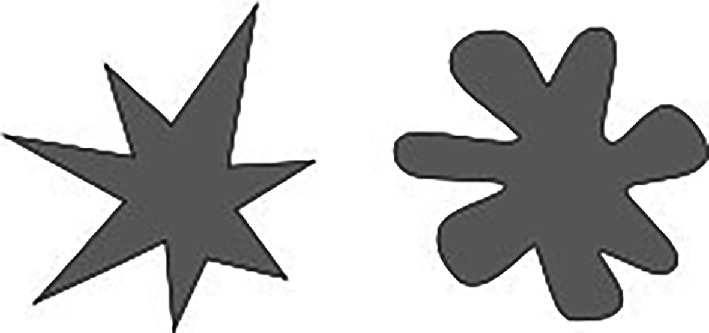
The jagged and rounded shapes used in bouba–kiki experiments [9].

The influence of iconicity on language has been further explored through experiments like the bouba-kiki task. In this task, participants are shown jagged and rounded figures (as seen in figure 1) and asked to match them with nonsense words such as ‘bouba’ and ‘kiki’. A majority of typical adults match ‘bouba’ with the rounded figure (on the right) and ‘kiki’ with the jagged figure (on the left), illustrating a natural propensity to link speech sounds with visual shapes. This effect, which is sometimes referred to as the ‘bouba–kiki’ effect, has been replicated in other languages [5], toddlers [6] and people from other cultures and writing systems as well [15,16].

Several hypotheses have been proposed to explain the bouba–kiki phenomenon. According to Ramachandran & Hubbard [17], participants are likely associating the visual features of the image (round/smooth versus spiky/sharp) with the articulatory gestures involved in producing the corresponding sounds. Specifically, the rounded lip posture and smooth inflections of the lips involved in producing the vowels in ‘bouba’ contrast with the retracted lip posture and sharp tongue and lip movements required to produce the vowels in ‘kiki’

It is also possible that such associations are based on the connections between pitch and shapes. Front vowels such as /i/ have higher frequencies (the second formant is much higher and is around 2500 Hz) when compared to back vowels (around 1200 Hz for /u/), and high-pitched sounds are generally associated with spiky shapes and low-pitched sounds are associated with round shapes [18]. Some studies have shown a stronger association between the consonants in these words and shapes. They showed that irrespective of the vowels, the consonants /b/, /m/, /l/ and /g/ were associated with ‘rounded’ shapes and the consonants /p/, t/, /k/ were associated with ‘angular’ or ‘spiky’ shapes [18–20]. The main difference between these two sets of sounds is that one set is voiced (/b/, /m/, /l/ and /g/ are produced with vocal fold vibrations) while the other set is voiceless (/p/, /t/ and /k/ are produced without any vocal fold vibrations). Voiced sounds have lower frequencies when compared to voiceless sounds and people generally associate low-pitched sounds with round shapes and high-pitched sounds with angular ones [18]. Some studies have also shown a link between orthography and the bouba–kiki phenomenon, especially among the literate adult population. That is, words containing angular letters (k) are matched with spiky shapes, and words containing rounded letters (b) are matched with rounded shapes [21]. However, given that this phenomenon is seen in blind individuals and people in the Himba tribe with no exposure to the Roman alphabet, it underscores the importance of the acoustic properties of sounds underlying this phenomenon [15,16]. Moreover, a recent study showed that the bouba–kiki effect is seen even for less pronounceable or unpronounceable sounds such as reversed words, sounds produced by objects, and pure tones. In all these cases, there seems to be a simple association between the pitch generated by these sounds and the shapes of referents [22]. Therefore, the participants seem to be making links between the acoustic or articulatory properties of the words/sounds and the visual contours. This phenomenon underscores the broader concept of crossmodal correspondences, highlighting how our sensory systems interconnect to shape perception and language learning.

### 1.2. The bouba–kiki effect on artificial word learning

Typically, in the bouba–kiki experiments, participants are presented with two distinct shapes—one rounded and one spiky—alongside two nonsense words, ‘bouba’ and ‘kiki’. When asked to match the nonsense words with the shapes, the participants can see the obvious differences between the shapes and the words and can easily make links between them (bouba–rounded; kiki–spiky) [19].

To address methodological limitations in prior research, Nielsen & Rendall [19] designed an experiment where they divided participants into two groups, presenting one group with a congruent pairing rule (curved objects matched with sonorous sounds like /m/, /n/ and jagged objects with plosives such as /p/, /t/) and the other with an incongruent pairing rule (curved objects matched with plosives, and jagged objects with sonorants). In subsequent test trials, where participants were asked to determine whether the image–word pairs conformed to the learned rules, those in the congruent condition outperformed those in the incongruent condition, by a small but significant margin (53.3% versus 50.4% correct, respectively, *t* (46) = 2.4, *p* < 0.01).

A study by Aveyard [23] used a word learning paradigm where participants were auditorily presented with the target words containing either plosives (words like *kuh-der-pai*) or non-plosives (words like *fuh-lih-sai*) and had to select one of the two objects/pictures (one rectilinear and one curvilinear). They were not provided with any guidelines for selecting the pictures that matched the words but were given feedback for their responses. In half of the trials, they were given positive feedback for congruent matching responses (plosives matched with rectilinear and non-plosives matched with curvilinear), and in the other half, positive feedback was provided for incongruent matches. The participants’ improvement in word–picture matching was tracked over three rounds of testing. The test trials showed better performance in the congruent when compared to incongruent trials initially, indicating a natural inclination towards sound symbolism. However, an improvement was seen even for the incongruent trials in round 3 without any decline in accuracy on congruent trials indicating that the participants developed a non-sound-symbolic strategy over time.

To further test the robustness of sound-symbolic influences, a second experiment gave participants more options to choose from, making the task more complex and less reliant on sound-symbolic strategies. Even though the sound-symbolism effect was weak at first, it became much stronger in the third round of testing for some participants as they received negative feedback for the ineffective strategies they adopted in round 1. This suggests that sound symbolism can become the key strategy under the right conditions. Although the findings of the two experiments may seem slightly different on the surface, they both indicate the importance of sound symbolism strategies in word learning. However, it should be noted that unlike the traditional bouba–kiki tasks using forced-choice methods, using complex word learning tasks and/or increasing the number of choices during response can reduce the bouba–kiki effect.

The studies by Nielsen & Rendall [19] and Aveyard [23] presented target words auditorily and required participants to learn a rule (congruent or incongruent to the word meaning) based on the feedback received. Although the effects of sound symbolism or iconicity were weak in both these studies, they still found a significant advantage of sound symbolism in artificial word learning tasks. However, these studies did not use an explicit word learning paradigm wherein participants are directly exposed to novel words alongside their associated referents during a learning phase and later tested for comprehension in a testing phase. This methodology does not involve feedback during the learning phase and relies solely on a sound-symbolic learning strategy.

Novel word learning can be either implicit, which refers to incidental learning of words through context, or explicit, where individuals are directly exposed to arbitrary words. Although both types of word learning are important, explicit word learning is particularly dependent on the declarative memory system which has been implicated in the acquisition and use of words, bound morphemes and idioms. This process relies on structures in the medial temporal lobe including the hippocampus [24]. Given the importance of explicit word learning, the primary purpose of the current study was to explore the advantages of iconicity in novel word learning among healthy adults using an explicit word learning method, as outlined by Warren & Duff [25].

A recent large-scale cross-linguistic study demonstrated the robustness of the bouba–kiki phenomenon in speakers of 25 languages, representing 9 language families and 10 writing systems [16]. However, this study only used two words (bouba and kiki) and the participants had to select a shape corresponding to the word that was presented. While the bouba–kiki effect is cross-linguistically robust, explicit word learning contexts tend to be more challenging and may reduce the impact of iconicity. Therefore, the current study aimed to investigate how robust the bouba–kiki effect is among participants from four countries, each speaking different languages, different phonology and prosody, and distinct writing systems. Our data collection method and goals were similar to those of the study by Ćwiek *et al*. [16] in that the data collection involved an opportunity sample, and the goal was to assess the generalizability of the bouba–kiki effect across cultures, without making any specific predictions about any culture. By examining participants from diverse linguistic and cultural backgrounds, we sought to better understand the robustness of the bouba–kiki effect in explicit word-learning tasks.

The findings in this section highlight that sound symbolism can influence word learning, although its effects tend to diminish in more complex tasks and/or when the number of choices is increased during the testing phase of the word learning task. As we examine these variations, it is also crucial to explore how individual differences, particularly in individuals with autism spectrum disorders (ASD) or those with higher levels of autistic traits, may impact the bouba–kiki effect. In the next section, we will investigate how autism and autistic traits can influence the ability to make shape–sound associations, providing insights into how cognitive and developmental factors might modify the strength of this effect.

### 1.3. The relationship between autism and the bouba–kiki effect

Research that has used the bouba–kiki tasks, where participants had to make a connection between shapes and words, has shown a reduced effect in individuals with ASD. The pioneering study conducted by Oberman & Ramachandran [26] on 10 children with high-functioning autism and 20 age, sex, and IQ-matched controls showed that children with ASD made shape–word correspondences in the expected direction only 56% of the time when compared to 86% in the control group. In a follow-up study by Ocelli *et al*. [27], the ASD group was further divided into high- and low-functioning subgroups. Results showed a significant difference between them. While the high-functioning group showed a tendency to match shapes and sounds in a manner that was not identical to typical children, their results were still above chance. On the other hand, the low-functioning group’s results were not significantly different from chance, suggesting that there is a broader range of responses to these associations in this group.

Gold & Segal [28] investigated whether cognitive abilities or autistic symptoms had a greater impact on the bouba–kiki effect in individuals with ASD. The researchers discovered a significant negative correlation between autism quotient (AQ) scores and performance on the bouba–kiki task within the ASD group, meaning that higher AQ scores (more autistic traits) were associated with lower scores on the bouba–kiki task. This correlation was not observed in the typically developing control group, which was matched for age and sex. The authors concluded that the severity of autistic symptoms was the main factor explaining the reduced bouba–kiki effect in ASD participants. Król & Ferenc [29] found a correlation between nonverbal IQ and scores on a word–shape association task only in the ASD group. The level of autism also determined the scores on this task. In summary, the findings from all these studies showed a reduced bouba–kiki effect in children with ASD [26–29].

Several explanations have been proposed for this relationship, including a mirror neuron system deficit, which may impair the connection between auditory and sensory–motor systems, and the under-connectivity theory which suggests that weak neural connections may negatively affect the integration of visual and auditory stimuli. Additionally, the weak central coherence with enhanced perceptual functioning theory posits that individuals with ASD tend to focus more on details rather than overall patterns linking visual and auditory cues [26–29]. There appears to be a correlation between ASD and pattern perception. This ability to seek and recognize patterns is directly related to the heightened systemizing abilities of individuals with ASD, which allows them to analyse rule-based systems. Consequently, individuals with strong autistic traits may show a particular interest in science, technology, engineering, and mathematics (STEM)-related disciplines [30,31].

Chen *et al*. [32] have suggested that the bouba–kiki phenomenon is rooted in pattern perception. They explored the bouba–kiki effect through radial frequency (RF) patterns with varying frequency, amplitude, and spikiness. They discovered that the manipulation of RF pattern features influenced sound–shape matching. Participants were more likely to associate an RF pattern with ‘kiki’ when the frequency, amplitude and spikiness increased. Conversely, as these features decreased, participants were more likely to associate the pattern with ‘bouba’. Additionally, this study showed that participants’ associations shifted from ‘bouba’ to ‘kiki’ as the visual characteristics of a pattern gradually changed across three dimensions, with the shift occurring as the level of spikiness in the visual features increased. In a follow-up study [33], Chen *et al*. reinforced the idea that the bouba–kiki effect is driven by a shared process in which participants extract key visual features—such as frequency, amplitude and spikiness—and apply crossmodal correspondence rules to match these features with sounds. These findings highlight that sound–shape associations are based on how visual patterns are processed and matched with corresponding sounds.

The enhanced pattern perception of individuals with ASD might enable them to extract visual features of shapes and then match them to acoustic properties of sounds as required in bouba–kiki tasks. However, individuals with autism, who generally have enhanced pattern perception, show a reduced bouba–kiki effect. One possible explanation for this is that all these studies had small sample sizes. Since ASD is highly heterogeneous, the small sample sizes may not adequately capture the variability in their abilities. Additionally, the findings of a reduced bouba–kiki effect could be affected by other bottom-up factors such as auditory and language processing in individuals with ASD.

Furthermore, autistic traits can be measured in the general population, yet the connection between autistic traits and word-learning tasks based on the bouba–kiki phenomenon has not been explored. While some studies have investigated the relationship between crossmodal association (such as colour–shape, colour–taste and shape–taste) abilities and autistic traits in typical adults [34,35], the specific relationship between the bouba–kiki effect and autistic traits in typical adults, without a diagnosis of ASD, has not been explored. Therefore, the second purpose of the current study was to investigate the association between autistic traits and the bouba–kiki-based word learning task within the general population with no diagnosis of autism. By conducting this study with a larger sample size, we can circumvent the issues associated with small sample sizes often seen in studies focused on individuals with ASD, as well as address potential confounding factors related to auditory and language processing that may affect results in this population. While the reduced bouba–kiki effect may be linked to deficits in the mirror neuron system, under-connectivity and weak central coherence, the current study seeks to explore whether pattern recognition, as observed in individuals with high levels of autistic traits, could play a role in this phenomenon. Moreover, given that autistic traits can vary across cultures, with higher levels observed in Eastern compared to Western cultures [36], we also aim to explore the influence of cultural context on the relationship between autistic traits and performance on a bouba–kiki-based word-learning task in both Western and Eastern populations.

## 2. Material and methods

### 2.1. Participants

#### 2.1.1. Brazil

Two hundred and sixty one speakers of Brazilian Portuguese, residing in Brazil (males = 81, females = 180) ranging in age from 18 to 40 years (mean = 27.0, s.d. = 7.04) who were generally healthy with no reported history of neurological/psychological issues, learning disability, hearing, and visual problems (normal or corrected-to-normal vision) participated in the study.

#### 2.1.2. India

Four hundred and sixteen multilingual speakers of various Indian languages, residing in India (males = 292, females = 124) ranging in age from 18 to 34 years (mean = 21.1, s.d. = 1.46) who were generally healthy with no reported history of neurological/psychological issues, learning disability, hearing and visual problems (normal or corrected-to-normal vision) participated in the study. Additionally, information about their academic majors and languages spoken was also collected.

#### 2.1.3. Japan

Four hundred and ninty three native speakers of Japanese, residing in Japan (males = 301, females = 181, prefer not to answer = 11) ranging in age from 18 to 40 years (mean = 33.2, s.d. = 5.91) who were generally healthy with no reported history of neurological/psychological issues, learning disability, hearing and visual problems (normal or corrected-to-normal vision) participated in the study.

#### 2.1.4. United States

Three hundred and eleven native speakers of American English, residing in the United States (males = 141, females = 170) ranging in age from 18 to 40 years (mean = 28.7, s.d. = 6.93) who were generally healthy with no reported history of neurological/psychological issues, learning disability, hearing and visual problems (normal or corrected-to-normal vision) participated in the study.

### 2.2. Stimuli

Pictures and audio recordings of novel names to go with the pictures were used for the congruent and incongruent phases of the experiment. The name–picture pairs included in the congruent condition were: bamoo (rounded), kuhtay (sharp/spiky), gogaa (rounded), teetay (sharp/spiky), noboma (rounded), and chitiki (sharp/spiky). The name–picture pairs included in the incongruent condition were: booba (sharp/spiky), kaykee (rounded), mabuma (sharp/spiky), tuhkeetee (rounded), keeji (rounded) and huhu (sharp/spiky). The words and pictures used in the current study were adapted based on the stimuli used by Maurer *et al*. [6] and Passi & Arun [22]. All pictures were drawn by a professional artist, and the auditory stimuli were recorded by a female speaker of American English using the Audacity software.

### 2.3. Procedure

The studies were conducted online using a Web-based survey platform, Qualtrics. Participants were recruited through the authors’ universities and Centiment survey panels (only for the US population). The panel participants received compensation from Centiment. The Qualtrics survey began with informed consent and questions related to demographics, languages spoken and health. This was followed by a brief experiment and a questionnaire. All participants read and agreed to the informed consent before starting the survey. The entire survey took about 10−15 min to complete.

**Figure 2.**
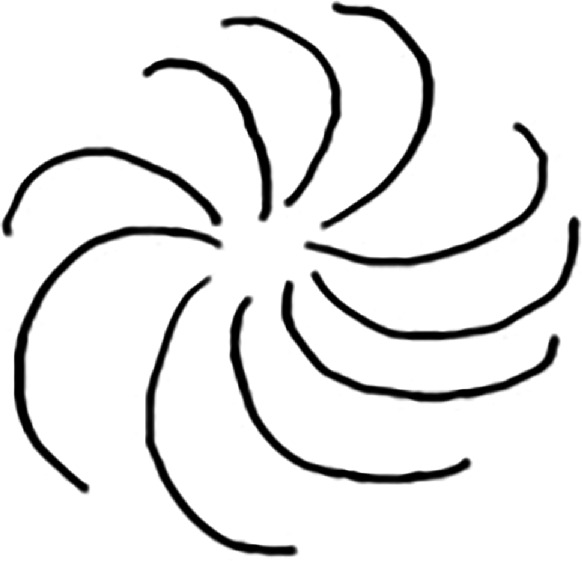
Learning phase: this is bamoo (congruent).

**Figure 3.**
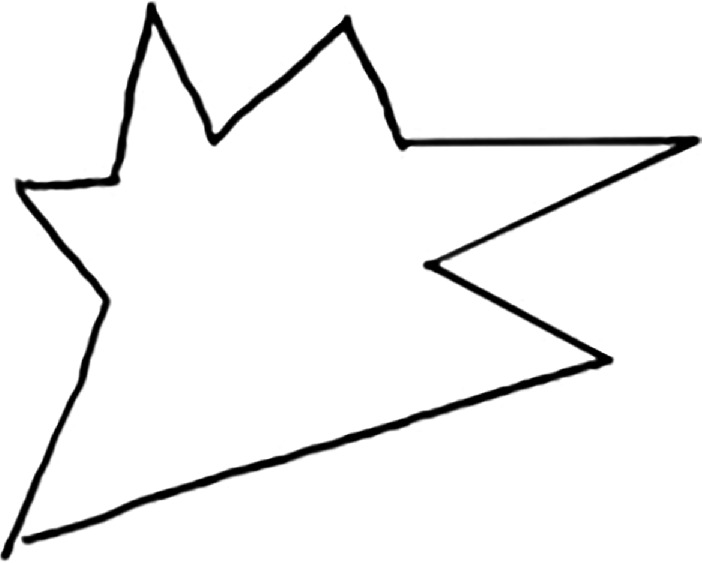
Learning phase: this is booba (incongruent).

The word learning experiment was adapted from a study by Warren & Duff [25]. There were two phases in this within-subject word learning experiment: a learning phase followed by a test phase. In the learning phase, the participants were presented with a novel unusual image (either rounded or spiky) accompanied by an audio recording of its name (female voice). There was a total of 12 novel images presented during the learning phase. Half (six) of these images were given congruent names, and the other half were given incongruent names. In the congruent picture–name pairs, there was a sound-symbolic (iconic) match between the pictures and the words, and in the incongruent picture–name pairs, there was no such association. For example, in the congruent pairs, a rounded image was paired with a word like ‘bamoo’ which had a rounded vowel and a voiced consonant /b/. In the incongruent pairs, a sharp image was paired with a word like ‘booba’ which had a rounded vowel /u/ and a voiced consonant /b/, which in this case was a mismatch. The presentation of the stimuli in this phase was randomized. Figure 2 depicts the presentation of the stimuli ‘bamoo’ in the learning phase (a congruent pair) and figure 3 depicts the presentation of the stimuli ‘booba’ in the learning phase (incongruent pair).

**Figure 4.**
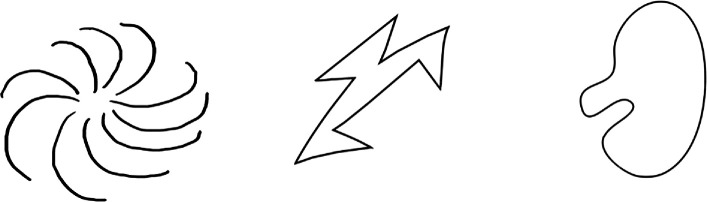
Test phase: where is bamoo? (congruent).

**Figure 5.**
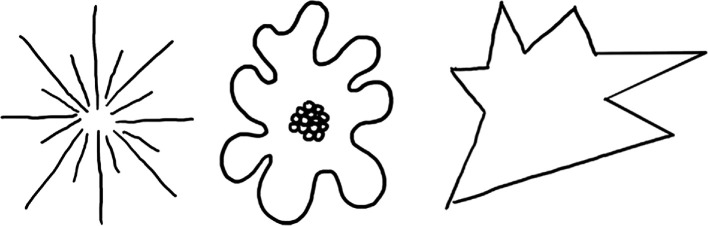
Test phase: where is booba? (incongruent).

The learning phase was followed by a test phase which used a three-alternative forced choice (3AFC) format (see figures 4 and 5). In this phase, the participants had to select a target picture from an array of three pictures; one picture in the array represented the target, while the other two pictures were the ones they had seen during the learning phase but were not the target. A unique array was presented 12 times to test each of the 12 target pictures that the participants had learned. The participants were asked in an audio recording ‘Where is (target name here)’, and expected to select one of the three images before moving on to the next array. The presentation of the stimuli in the test phase was randomized. The participants did not receive any feedback during the learning or test phases of the study.

The learning of the 12 novel items and their testing was preceded by a practice trial where two novel word–picture pairs were presented and tested with feedback. The written instructions for informed consent, practice, learning and test phases were provided in English for the US and Indian participants, in Portuguese for the Brazilian participants, and in Japanese for the Japanese participants. The audio portion of the survey, which included the presentation of the stimuli during the learning phase (e.g. ‘This is bamoo’) and the target (e.g. ‘Where is bamoo?’) during the testing phase was in American English for participants from all countries. This was done to maintain consistency in the pronunciation of the stimuli. Moreover, all participants were well-versed in English, and the statements used during the presentation of the stimuli and target were simple and easy to understand.

Following the word learning phase, the survey included a final section to determine the degree of autistic traits among participants. In this phase, participants completed the AQ10 [37,38], which measures the degree of autistic traits. This questionnaire is widely used in research and clinical practice to measure the degree of autistic traits. It was first developed as a self-reported questionnaire and later developed as a parent-reported questionnaire for adolescents and children. The AQ10 has been validated in Brazil, Japan, and the USA but not in India. However, a few studies have used the full AQ English version in India [36]. They were asked to read a series of statements regarding personality, personal habits, and preferences, and select the response that best applied to them. For example, participants were presented with statements such as ‘I find it easy to do more than one thing at once’ and ‘I know how to tell if someone listening to me is getting bored’. Response options included ‘definitely agree’, ‘slightly agree’, ‘slightly disagree’, or ‘definitely disagree’. Participants were required to respond to a total of ten statements. Based on their responses, participants received a score on a scale from 0 to 10; 0 indicates little to no autistic traits, whereas 10 indicates the presence of high levels of autistic traits.

## 3. Results

### 3.1. Bouba–kiki effect

Overall, a paired sample *t*‐test (two-tailed) revealed a significant difference between the congruent and incongruent word recognition scores across all four countries (*t* (260) = 2.67, *p* = 0.008, *d* = 0.18, 95% CI[0.07, 0.47] for Brazil, *t* (415) = 5.75, *p* < 0.001, *d* = 0.31, 95% CI[0.30, 0.62] for India, *t* (492) = 5.73, *p* < 0.001, *d* = 0.30, 95% CI[0.29, 0.59] for Japan and *t* (310) = 3.65, *p* < 0.001, *d* = 0.25, 95% CI[0.16, 0.53] for USA). This shows that the bouba–kiki effect was robust across all four countries.

**Figure 6.**
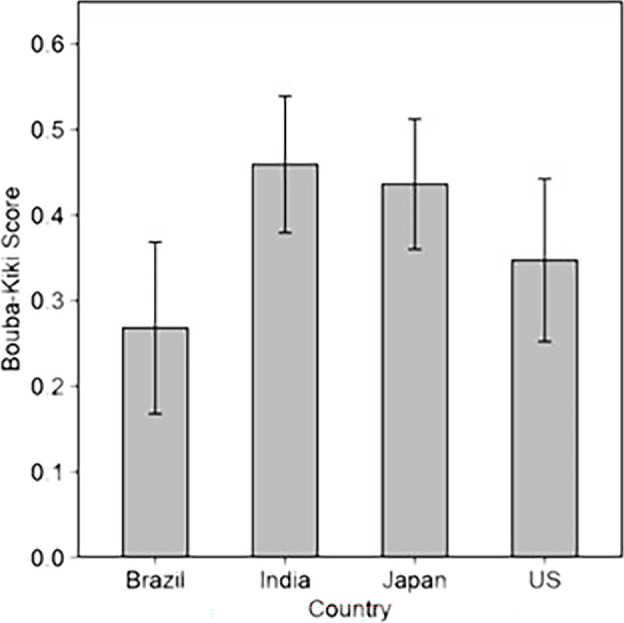
Average bouba–kiki scores across countries. Error bars represent the standard errors.

**Figure 7.**
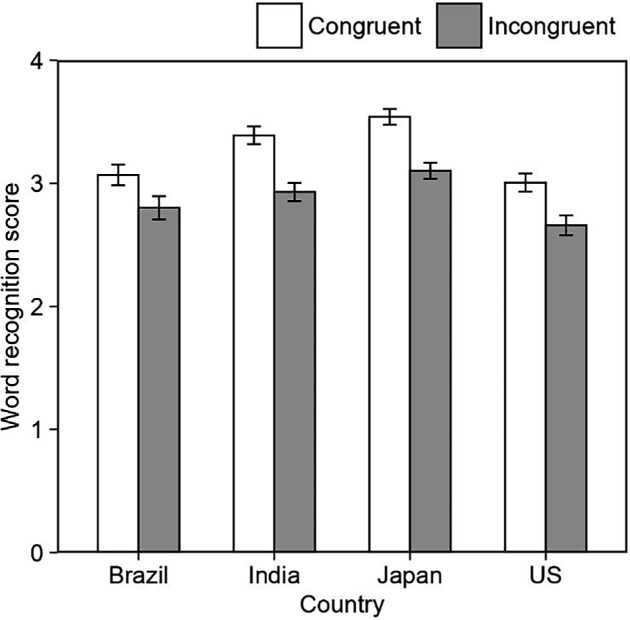
The proportion of congruent and incongruent word recognition scores across countries.

A bouba–kiki score was calculated for each participant by subtracting the incongruent word recognition score from the congruent word recognition score. For example, during the testing phase, if a participant correctly recognized 5 out of the 6 pictures presented from the congruent condition (congruent word recognition score = 5), and 4 out of the 6 pictures from the incongruent condition (incongruent word recognition score = 4), then this participant’s bouba–kiki score would be 1 (congruent − incongruent score). An average of this score was calculated for each country to obtain the mean bouba–kiki score for all four countries (see figure 6). Figure 7 shows the proportion of congruent and incongruent word recognition scores of participants across countries. A one-way ANOVA was performed to compare the strength of the bouba–kiki effect across all four countries. The results showed no difference in the strength of the bouba–kiki effect across countries (*F* (3, 1477) = [0.90], *p* = 0.439, *η*_G_^2^ = 0.00).

### 3.2. Association between autism quotient and the bouba–kiki effect

**Figure 8.**
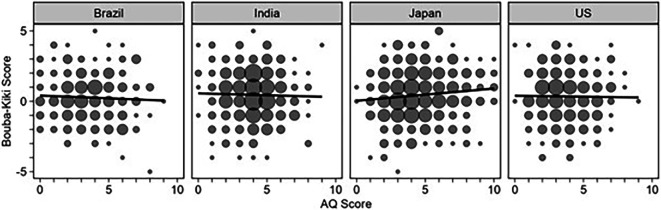
Correlation between the bouba–kiki and AQ scores across countries.

A Pearson’s correlation between AQ and the mean bouba–kiki score was calculated for each country (see figure 8). We found no significant correlation between the AQ score and the bouba–kiki score using a two-tailed test for Brazil (*t* (259) = −0.76, *r* = −0.05, *p* = 0.449, 95% CI[−0.17, 0.07]), India (*t* (414) = −0.53, *r* = −0.03, *p* = 0.60, 95% CI[−0.12, 0.07]), and the USA (*t* (309) = −0.24, *r* = −0.01, *p* = 0.814, 95% CI[−0.12, 0.10]). However, a weak but significant positive correlation was found between AQ and the bouba–kiki score for the Japanese participants (*t* (491) = 2.34, *r* = 0.11, *p* = 0.020, 95% CI [0.02, 0.19]).

The overall effect of the AQ score on the bouba–kiki score was examined using a iear mixed model (LMM) analysis in R with the *lme4* package [39] and the *lmerTest* package [40]. Initially, the model was fitted by including the country factor as both a random intercept and a random slope, using restricted maximum likelihood (REML) for parameter estimation (REML criterion at convergence = 5708.4; Akaike information criterion (AIC) = 5720.4). However, a singular fit was observed, as indicated by the isSingular() function, suggesting that the random effect variance for the country factor was essentially zero. To address this, the model was re-specified to include only the random slope for the country factor, which resolved the singular fit issue. The final model fit was assessed using REML, yielding a criterion at convergence of 5709.5 and an AIC of 5717.5. The fixed effect of AQ scores was found to be non-significant (estimate = 0.021, s.e. = 0.024, *t* = 0.87, *p* = 0.392, 95% CI [−0.020, 0.070]), suggesting that the AQ scores did not significantly predict bouba–kiki scores, as shown in table 1.

**Table 1.** Linear mixed model results showing the overall effects of AQ on bouba–kiki scores. Number of observations = 1481; s.e. = standard error; s.d. = standard deviation; CI = confidence interval; LL = lower limit; UL = upper limit; AQ = autism quotient.

effect	estimate	s.e.	s.d.	*t*	*p*	95% CI	
						LL	UL
fixed effect							
(intercept)	0.301	0.103		2.93	0.003	0.092	0.494
AQ	0.021	0.024		0.87	0.392	−0.020	0.070
random effect							
country–AQ			0.012			0.000	0.046
residual			1.658			1.599	1.719

### 3.3. The strength of autistic traits across countries

**Figure 9.**
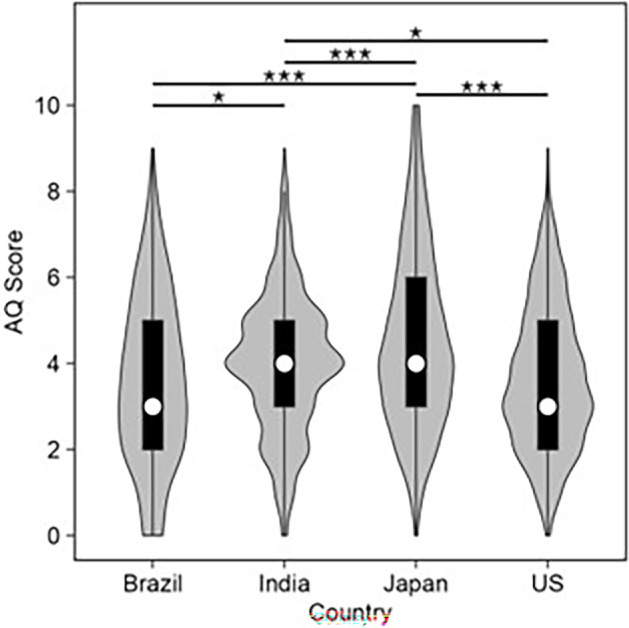
The strength of AQ score across countries: Japan > India > Brazil ≒ USA. Asterisks at the top of the figure show the degree of statistical difference (*p < 0.05, **p < 0.01, ***p < 0.001).

An ANOVA of the AQ scores shown in figure 9 revealed a main effect of the country: *F*(3, 1477) = 21.7, *p* < 0.001, *η*_G_^2^ = 0.04. Multiple comparison for main effect of country showed that Brazil < India: *t*(1477) = 2.67, *p*_holm_ = 0.023; Brazil < Japan: *t*(1477) = 6.70, *p*_holm_ < 0.001; Brazil ≒ USA: *t*(1477) = 0.42, *p*_holm_ = 0.674; India < Japan: *t*(1477) = 4.53, *p*_holm_ < 0.001; India > USA: *t*(1477) = 2.34, *p*_holm_ = 0.039; Japan > USA: *t*(1477) = 6.60, *p*_holm_ < 0.001. In summary, Japanese participants had the highest average AQ scores followed by the Indian participants. The Brazilian and US participants had similar average AQ scores, which were lower than those of the Indian participants.

## 4. Discussion

In this study, we aimed to examine the bouba–kiki effect in a word-learning context, building upon the approach used by Ćwiek *et al*. [16]. Both studies utilized an opportunity sampling method to assess the generalizability of the bouba–kiki effect across cultures, without making specific predictions about any one culture. By including participants from diverse linguistic and cultural backgrounds, we sought to deepen our understanding of the robustness of the bouba–kiki effect in explicit word-learning tasks. In addition to this, we explored whether autistic traits influenced word learning. We hypothesized that individuals with heightened pattern perception, often associated with higher levels of autistic traits, might be better able to form associations between the visual properties of shapes and the acoustic properties of sounds, as required in bouba–kiki tasks. The explicit word learning method used in the current study taps into pattern perception by pairing novel images with either congruent or incongruent names. In the congruent condition, where the word and image are conceptually aligned, participants are likely to rely on the visual features of the image and the phonetic properties of the word, mirroring the process of extracting key visual and auditory features observed in pattern perception studies.

The results revealed a significantly higher word recognition score in the congruent compared to the incongruent condition across all four countries. A one-way ANOVA showed no difference in the strength of the bouba–kiki effect between countries, indicating the robustness and universality of the effect. In other words, iconicity plays an important role in word learning among healthy adults, irrespective of their language, culture, or writing systems. These findings are consistent with a recent study by Ćwiek *et al*. [16], which demonstrated the robustness of the bouba–kiki effect across speakers of 25 languages and 10 writing systems. This study, however, used the forced-choice method where participants were presented with an angular and a rounded figure and asked to select one of them when auditorily presented with the words ‘bouba’ or ‘kiki’. The current study is the first to show the robustness of the bouba–kiki effect in an artificial word learning task among healthy adults across four countries. Moreover, we found the same effect among Indian participants, who collectively spoke 18 different languages in addition to English. It is important to note that some pseudowords used in the study could have phonological similarities with some of the languages tested. Furthermore, while recordings in American English may be perceived differently across populations, we chose to use them to ensure that any differences in findings could not be attributed to the stimuli themselves. Despite these potential limitations, the universality of the bouba–kiki effect on word learning remains noteworthy. These results are also consistent with findings that show the benefits of iconicity in artificial word learning tasks among typical adults [6,19,23]. Collectively, these studies suggest that adult word learners across different countries and languages are generally better at learning iconic associations between the acoustic properties of a word and the visual features of an image.

Only a few studies have explored the relationship between ASD and the bouba–kiki effect, and findings from all studies show a reduced effect. Moreover, the study by Gold & Segal [28] showed an association between scores on word–shape association tasks and degree of autistic traits, and the study by Król & Ferenc [29] showed an association between scores on the bouba–kiki task and nonverbal intelligence. The present study is the first one to investigate the relationship between autistic traits (measured by AQ) and the bouba–kiki effect (measured through the bouba–kiki score) in the general population using an explicit word learning task. First, an analysis of the strength of AQ across countries revealed that Japanese participants had the highest average AQ scores followed by the Indian participants. The average AQ scores of Brazilian and US participants were more similar and were lower than those of Indian participants. These findings are consistent with the findings by Freeth *et al*. [36] which showed higher levels of autistic traits among Eastern when compared to Western cultures.

A survey conducted in the UK on nearly half a million people showed higher AQ scores among people from STEM backgrounds when compared to their non-STEM counterparts [41]. In the current study, all the Indian participants were from an engineering college, and this can explain their relatively high AQ scores. Although the specific disciplines or occupations of participants from other countries are not known, it is possible that the Japanese participants had a greater proportion of STEM-related backgrounds when compared to the US and Brazilian participants. This could have potentially led to a higher average AQ among Japanese participants, but this cannot be confirmed without detailed information on their backgrounds. This, however, does not explain the higher AQ scores among Japanese when compared to the Indian participants who exclusively had STEM backgrounds. This suggests an interplay between culture and the field of participants in determining autistic traits.

An LMM analysis of the overall effect of AQ on the bouba–kiki scores showed that the AQ scores did not significantly predict bouba–kiki scores. This shows that the higher levels of systematicity and pattern recognition associated with AQ did not affect making associations between the acoustic properties of a word and the shapes of figures. Higher levels of autistic traits may be connected with visuospatial pattern perception tasks such as mental rotation and disembedding of figures/shapes from an array of geometric shapes [42,43] but not bouba–kiki-based word learning tasks. However, it should be noted that a correlation between AQ and the bouba–kiki scores for each country revealed a weak but significant positive correlation between the bouba–kiki effect and AQ only for the Japanese participants. This finding is consistent with a study that showed stronger colour–shape associations (e.g. circle–red, triangle–yellow) among Japanese adults with high autistic traits [34]. Given the weak association, this finding needs to be treated with caution and has to be explored further.

Overall, when the findings of the relationship between ASD and the bouba–kiki effect [26–29] and the results of the LMM analysis on the relationship between AQ and the bouba–kiki effect of the current study are combined, it is reasonable to conclude that having higher levels of autistic traits may not lead to an increase in the bouba–kiki effect. However, cultural factors could affect this relationship, which needs to be explored further. In addition to AQ, the relationship between bouba–kiki and other personality measures, such as systemizing quotient [44], which refers to the ability to understand the rules and patterns governing a system, may add more insight into the relationship between pattern perception and bouba–kiki tasks.

## 5. Clinical implications

The current study underscores the importance of iconicity in word learning. Iconic words are generally crossmodal and may be less susceptible to neurological damage [14]. If future research confirms that iconic associations are less vulnerable to damage, speech-language pathologists could incorporate these associations into their assessment toolkit and treatment strategies for naming and verbal fluency in acquired language disorders. This could include tasks where individuals make associations between nonsense words, such as ‘bouba’ and congruent shapes (rounded) and congruent tastes (sweet), which may lead to better memory and retention of words [45]. Support for this proposal comes from a recent study that shows that three-dimensional-printed flavour-based cues were effective in memory retrieval for healthy older adults because of their rich sensorial and emotional properties [46].

## Data Availability

The data have been made available online at OSF [47].
